# Validation of the Sleepiz One + as a radar-based sensor for contactless diagnosis of sleep apnea

**DOI:** 10.1007/s11325-024-03057-6

**Published:** 2024-05-14

**Authors:** Jonas Alexander Gross-Isselmann, Torsten Eggert, Alina Wildenauer, Sarah Dietz-Terjung, Martina Grosse Sundrup, Christoph Schoebel

**Affiliations:** Devision of Sleep & Telemedicine, Ruhrlandklinik, University Medicine Essen, University of Duisburg-Essen, Essen, Germany

**Keywords:** OSA, Digitalization, High-frequency electromagnetic fields, Sleep monitoring, Diagnosis, Apnea

## Abstract

**Purpose:**

The cardiorespiratory polysomnography (PSG) is an expensive and limited resource. The Sleepiz One + is a novel radar-based contactless monitoring device that can be used e.g. for longitudinal detection of nocturnal respiratory events. The present study aimed to compare the performance of the Sleepiz One + device to the PSG regarding the accuracy of apnea–hypopnea index (AHI).

**Methods:**

From January to December 2021, a total of 141 adult volunteers who were either suspected of having sleep apnea or who were healthy sleepers took part in a sleep study. This examination served to validate the Sleepiz One + device in the presence and absence of additional SpO2 information. The AHI determined by the Sleepiz One + monitor was estimated automatically and compared with the AHI derived from manual PSG scoring.

**Results:**

The correlation between the Sleepiz-AHI and the PSG-AHI with and without additional SpO2 measurement was r_p_ = 0.94 and r_p_ = 0,87, respectively. In general, the Bland–Altman plots showed good agreement between the two methods of AHI measurement, though their deviations became larger with increasing sleep-disordered breathing. Sensitivity and specificity for recordings without additional SpO2 was 85% and 88%, respectively. Adding a SpO2 sensor increased the sensitivity to 88% and the specificity to 98%.

**Conclusion:**

The Sleepiz One + device is a valid diagnostic tool for patients with moderate to severe OSA. It can also be easily used in the home environment and is therefore beneficial for e.g. immobile and infectious patients.

**Trial registration number and date of registration for prospectively registered trials:**

This study was registered on clinicaltrials.gov (NCT04670848) on 2020–12-09.

**Supplementary Information:**

The online version contains supplementary material available at 10.1007/s11325-024-03057-6.

## Introduction

Obstructive sleep apnea (OSA) is a common chronic sleep disorder with prevalences for mild to severe OSA of up to 38% [1]. A more recent literature-based analysis even suggests that around one billion adults worldwide could be afflicted with OSA, with prevalences of more than 50% in some countries [2]. Growing numbers can be explained by improvements in diagnostic standards for OSA [3], and by the global rise of two important OSA risk factors: the steadily increase in obesity [4, 5] and life expectancy [6]. Despite substantial consequences on health, undiagnosed OSA is far more common than expected. A study from 1997 observed that about 80–90% of patients with moderate to severe OSA remained undetected [7]. Undiagnosed OSA may especially affect patients without typical risk factors (male sex, old age and obesity) and/or comorbidities (hypertension) [8]. At the same time, insufficient knowledge about heterogeneous clinical manifestations of OSA among healthcare professionals also carries the risk that patients suffering from e.g. excessive daytime sleepiness (EDS) are more likely to go undiagnosed [9]. Another reason for the discrepancy between prevalence and diagnosis rates are long waiting times for standard sleep examinations such as cardiorespiratory polysomnography (PSG) due to overall limited availability [10]. To address these challenges adequately, further capacities such as non-contact, ambulatory diagnostic procedures that are user-friendly and accurate are urgently needed. Currently, a few devices designed for non-contact monitoring in ambulatory settings using different technologies exist yet have not been introduced into routine care. The radar-based Res Med SleepMinder detects sleep-disordered breathing (SDB) with high diagnostic accuracy [11]. Nocturnal respiratory events can also be detected by camera-based sensors. In a small study with three patients, a classification accuracy of 82% was achieved [12]. The Withings sleep analyzer is an under-the-mattress device, which measures movements of the body and chest (breathing), and vibrations due to cardiac ejection (ballistocardiography) [13]. Another under-the-mattress approach is the recently published method for detecting respiration patterns via sensors placed between the mattress and bed frame, employing a modified U-Net analysis model for OSA detection, achieving 76.4% in sensitivity [14]. In addition to contactless measuring, there are low-contact devices, which operate with minimal body contact, like the Sunrise system RDI, which is attached to the patients’ chin measuring mandibular movements [15]. Another approach is to bring PSG diagnostics home. A study involving 960 participants to assess home-based self-applied polysomnography demonstrated successful recording in 88.6% of cases [16].

The present study focuses on the Sleepiz One + device, a system based on the Doppler Effect that uses electromagnetic wave reflections to measure small body movements, such as those generated by heartbeat or respiration [17–19]. Since electromagnetic waves at certain frequencies are largely unaffected by clothes, bedsheets, or room acoustics, radar technology is specifically suited for measurements in the home environment, where the patient is usually covered by a blanket, sleeps in the same bed with a partner and is exposed to some background noise during the night. Although radar-based approaches to monitor a person's sleep have been well established and several sensors based on this technology have already been validated [20], none of these systems were implemented into clinical routine. Aim of this work was to determine how well the Sleepiz One + device performs in detecting sleep-disordered breathing compared to the diagnostic gold standard, the cardiorespiratory PSG.

## Methods

### Study participants

A total of 141 adult participants including 120 patients (69 males: mean age 53.5 years ± 15.8 (SD) years, 22–88 years; 51 females: 57.1 years ± 13.8 years, 22–89 years) with suspected OSA and 21 healthy volunteers (15 males: 43.5 years ± 16.7 years, 19–73 years; 6 females: 34 years ± 12.1 years, 24–55 years) had been enrolled within an eleven-month period (first participant: 28.01.2021; last participant: 20.12.2021). Potential participants had been asked to take part in the study during their regular OSA diagnostic appointment at the clinic (Ruhrlandklinik, Essen, Germany), or were recruited by word-of-mouth recommendation. Exclusion criteria were cardiac pacemaker or other implanted electrical devices, pregnancy or lactation, and patients who were unable to consent. A detailed list of inclusion and exclusion criteria is attached in the appendix. Healthy volunteers were exclusively recruited through word of mouth. The healthy volunteers received appropriate monetary compensation for their participation, while it was assumed that patients might benefit from this new diagnostic method in the future. The study followed the principles of the Declaration of Helsinki, complied with the European Medical Device Regulation (MDR, EUDAMED number: CIV-20–08-034466), and was approved by the Ethics Committee Essen (20–9657-MF). All individuals signed an informed consent prior to participation.

### Study procedure

Participants were scheduled to undergo a cardiorespiratory PSG according to the standard of the American Academy of Sleep Medicine (AASM) [21] to determine the presence and/or the severity of OSA using the Nox A1 PSG System (Nox Medical, Reykjavik, Iceland). PSG monitoring lasted at least 6 h and were performed in parallel with the Sleepiz One + recording.

The apnea hypopnea index (AHI) was manually scored by three independent sleep technicians according to the current scoring rules of the AASM, with hypopneas being scored according to AASM Scoring Manual Version 2.6. Apnea and hypopnea events during sleep were extracted from the PSG data and categorized (AHI < 15; AHI ≥ 15). If the three scorers did not unanimously agree on one of the two categories, the data was excluded from further analysis. Otherwise, the severity category chosen was considered the ground truth. The Sleepiz One + recordings were analyzed automatically using the latest version of the scoring algorithm. This computation was performed twice, once with and once without additional information on arterial oxygen saturation from a pulse oximeter.

Recordings belonging together were synchronized by using proprietary software and visualized to evaluate the quality of the synchronization. The time offset between the two data files was adjusted if necessary. The signals from the PSG were then visually assessed to identify artifacts or time intervals of low signal quality, which were marked and removed from further analysis. Signals from the Sleepiz One + recording were also processed with proprietary software for identification and deletion of motion artifacts and time intervals of low signal quality. If more than 50% of the recording had to be removed, the entire measurement of this particular participant was excluded from the analysis.

### Non-contact monitoring using Sleepiz One + 

**Fig. 1:**
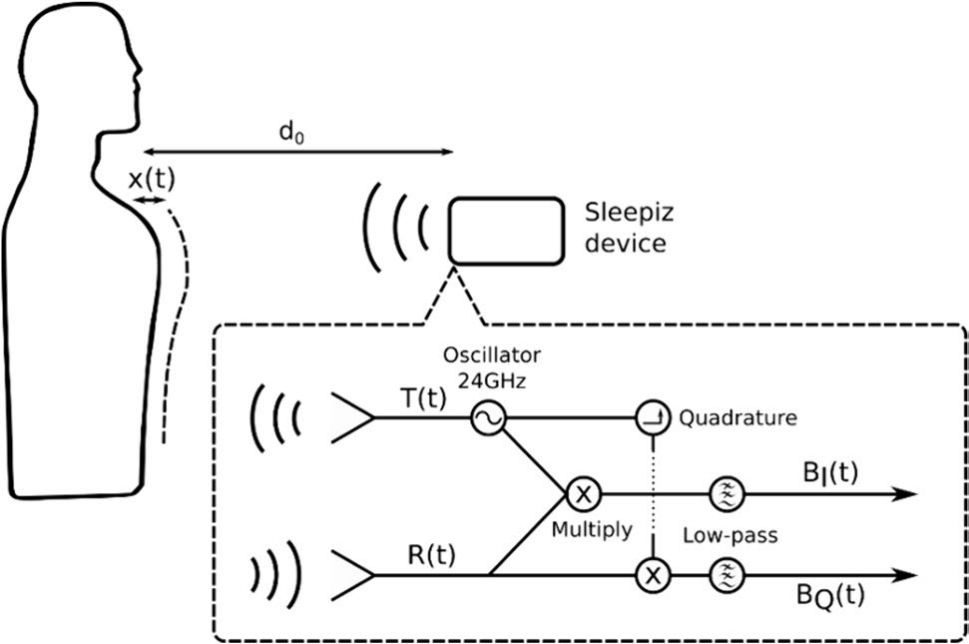
Schematic of radar operation. T(t) transmitted electromagnetic signal (f = 24 GHz), R(t) reflected signal, BI(t) in-phase channel, received signal multiplied by transmitted signal, low-pass filtered, BQ(t)quadrature channel, received signal multiplied by 90° offset of transmitted channel, low-pass filtered. Figure provided by Sleepiz AG, Zürich, Switzerland; appeared in unpublished poster related to [33]. It was later published in 2023 by Bujan et al. in the article “Clinical validation of a contactless respiration rate monitor” [24]

The Sleepiz One + hardware unit uses radar technology to allow contactless monitoring of breathing patterns, respiration rate and heart rate at rest or during sleep. It is mounted on a stand beside the patient’s bed, slightly elevated above mattress level and approximately 50 cm from the patient's thorax. From that position, the Sleepiz radar sensor detects object distance changes by transmitting fixed-frequency electromagnetic waves, collecting reflected signals, and processing them to obtain the analog output signals BI(t) and BQ(t). These data streams indicate relative distance changes caused by thorax and abdomen movements [22], mimicking the summed respiratory inductance plethysmography signal that can be used in PSG to detect apnea and hypopnea when the recommended airflow detection device fails [23]. The sensor prioritizes objects with the largest radar cross-section and filters out static background clutter. The transmitter emits electromagnetic waves (24 GHz) with a defined beam aperture (80°/34°), which are then reflected to the transceiver. Received signals are amplified, multiplied by the transmitted signal (I-channel) and a 90° offset version (Q-channel), and eventually down-converted to an intermediate frequency (B(t)). After the completion of these operations, the information contained in the signal, B(t), is the change of phase of the transmitted signal after being reflected and arriving back to the transceiver. The resulting information in B(t) represents the phase change of the transmitted signal after reflection [24]. Figure 1 illustrates a schematic depiction of the operational mode. By recognizing typical movement patterns, a classification is made to determine the presence or absence of a respiratory event, and to distinguish between wakefulness and sleep. The AHI is then calculated based on these measurements. The parameters can be visualized with a web application to which the data is transferred using Wi-Fi connectivity. The intended purpose of the Sleepiz One + web application is to display and analyze data sent by the Sleepiz One + hardware unit. The web application allows the analysis and annotation of the data, compilation of results into reports and the management of the Sleepiz One + hardware units.

### Statistical analysis methods

We defined an acceptable value of type I error probability (α) of 0.05, and a type II error probability (β) of 0.1. Furthermore, we assumed a prevalence of 50% within our study population of subjects with AHI greater than or equal to 15, a minimum effect size of 5%, i.e. a minimum acceptable sensitivity and specificity of 70%, and, for the power calculation, a model specificity and sensitivity of 80%. We based our sample size calculation on Monte Carlo simulations of the task at hand and selected the minimal sample size needed that satisfied our initial criteria: α ≤ 0.05, and β ≤ 0.1.

The minimal required sample size was 105 study participants (α = 0.050, and β = 0.084). Adjusting for an estimated data loss of 10%, we aimed at enrolling 116 participants. Data loss of 10% was expected due to the possibility of misplacement of electrodes during PSG recording or to suboptimal positioning of the Sleepiz device since both scenarios would result in insufficient data quality.

The primary outcome of interest was the diagnostic testing performance of the Sleepiz device One + based on a binary classification of OSA requiring treatment independently of symptoms or comorbidities (AHI ≥ 15) and no or mild OSA not mandatorily requiring treatment (AHI < 15). Its validity was determined by sensitivity and specificity measures. Secondary outcome was the relationship between the two AHI measurement methods, examined by means of Pearson correlation and Bland–Altman analyses. Bland- Altman analysis was chosen in order to provide a comprehensive assessment of the agreement between the two measurement methods. It is an established method in comparative analysis in the medical field and proved to be a more appropriate approach than linear regression in this context [25]. Bland–Altman analysis computes the difference between values obtained by two distinct methodologies (of which one is considered as the gold standard) and plotting them against their respective means. This technique closely resembles residual analysis in regression and can be used to uncover any systematic, proportional, and random bias in the data. The presence of statistically significant systematic error was tested by means of a one sample t-test using zero as reference, while Pearson correlation analysis was applied to identify statistically significant proportional error between the two methods. This is particularly crucial for ensuring the accuracy and reliability of diagnostic procedures. On the basis of this information, conclusions were drawn as to whether the two methods examined are basically interchangeable. [26]. The results of continuous variables were depicted as mean and standard deviation (SD), categorical variables were expressed as numbers (%).

## Results

Overall, 28 data sets had to be excluded due to technical issues. An additional of four recordings had to be disregarded because of very short signal duration (< 4000 secs), reference thorax channel missing, no overlap between reference and Sleepiz data, and poor signal quality in the Sleepiz One + recording. A discrepancy between the manual annotators of the PSG data occurred in nine cases. These nine recordings were also not considered for further analysis, resulting in a final sample of 100 subjects. Demographics of the study population are presented in Table 1.

**Table 1:** Demographics of the study participants

n = 100	Age (years) Mean ± SD	BMI (kg/m2) Mean ± SD	AHI [PSG] Mean ± SD	AHI [Sleepiz] Mean ± SD	AHI [Sleepiz & SpO2] Mean ± SD
Male (n = 57)	51.42 ± 16.99	29.15 ± 6.57	18.51 ± 17.59	20.76 ± 16.22	16.03 ± 16.49
Female (n = 43)	52.74 ± 16.58	30.14 ± 7.89	13.58 ± 16.04	12.78 ± 12.65	10.90 ± 14.28

Values are given as mean and standard deviation (SD). BMI: Body Mass Index; SpO2: Peripheral oxygen saturation. *AHI* Apnea Hypopnea Index, *PSG* Polysomnography

### Clinical test performance based on binary sleep apnea severity classification

The Sleepiz algorithm without SpO2 correctly classified clinically confirmed patients suffering from moderate to severe sleep apnea (AHI ≥ 15) with 85%. In addition, individuals with mild or no sleep apnea were identified as such with a probability of 88%. The positive and negative predictive values of this Sleepiz algorithm were 83% and 90%, respectively. By adding the pulse oximeter, a sensitivity of almost 88% was achieved. The improvement in specificity attributable to the pulse oximeter was even higher. The probability of identifying a healthy person rose to over 98% (see Table 2 and 3). Positive and negative predictive values improved to 97% and 93%, respectively.

**Table 2:** Apnea severity classification performance of the Sleepiz One + monitor

n = 100	PSG AHI ≥ 15	PSG AHI < 15	
Sleepiz AHI ≥ 15	35	7	PPV = 83.3%
Sleepiz AHI < 15	6	52	NPV = 89.7%
	sensitivity = 85.4%	specificity = 88.1%	

*PPV* Positive predictive value, *NPV* Negative predictive value, *AHI* Apnea Hypopnea Index, *PSG* Polysomnography

**Table 3:** Apnea severity classification performance of the Sleepiz One + monitor including an additional SpO2 sensor

n = 100	PSG AHI ≥ 15	PSG AHI < 15	
Sleepiz SpO2 AHI ≥ 15	36	1	PPV = 97.3%
Sleepiz SpO2 AHI < 15	5	58	NPV = 92.1%
	sensitivity = 87.8%	specificity = 98.3%	

*PPV* Positive predictive value, *NPV* Negative predictive value, *AHI* Apnea Hypopnea Index, *PSG* Polysomnography, *SpO2* Peripheral oxygen saturation

### Relationship between two AHI measurement methods

**Fig. 2:**
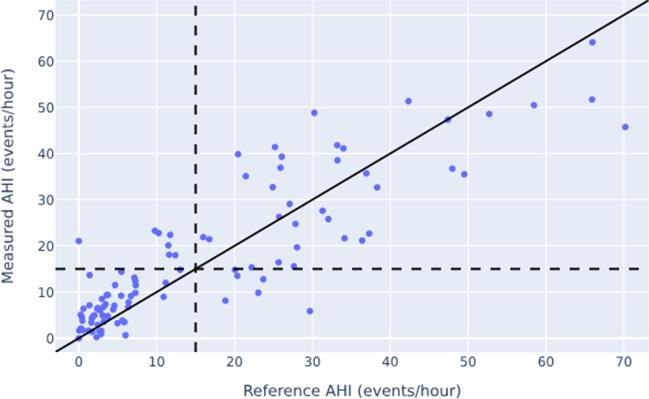
Relationship between the AHI measured by means of the Sleepiz One + monitor and the reference AHI obtained using the PSG (Please note that the black line displays the line of perfect agreement). Pearson Correlation Coefficient was r_p_ = 0.87 (*p* < 0.0001). AHI: Apnea Hypopnea Index; PSG: Polysomnography

**Fig. 3:**
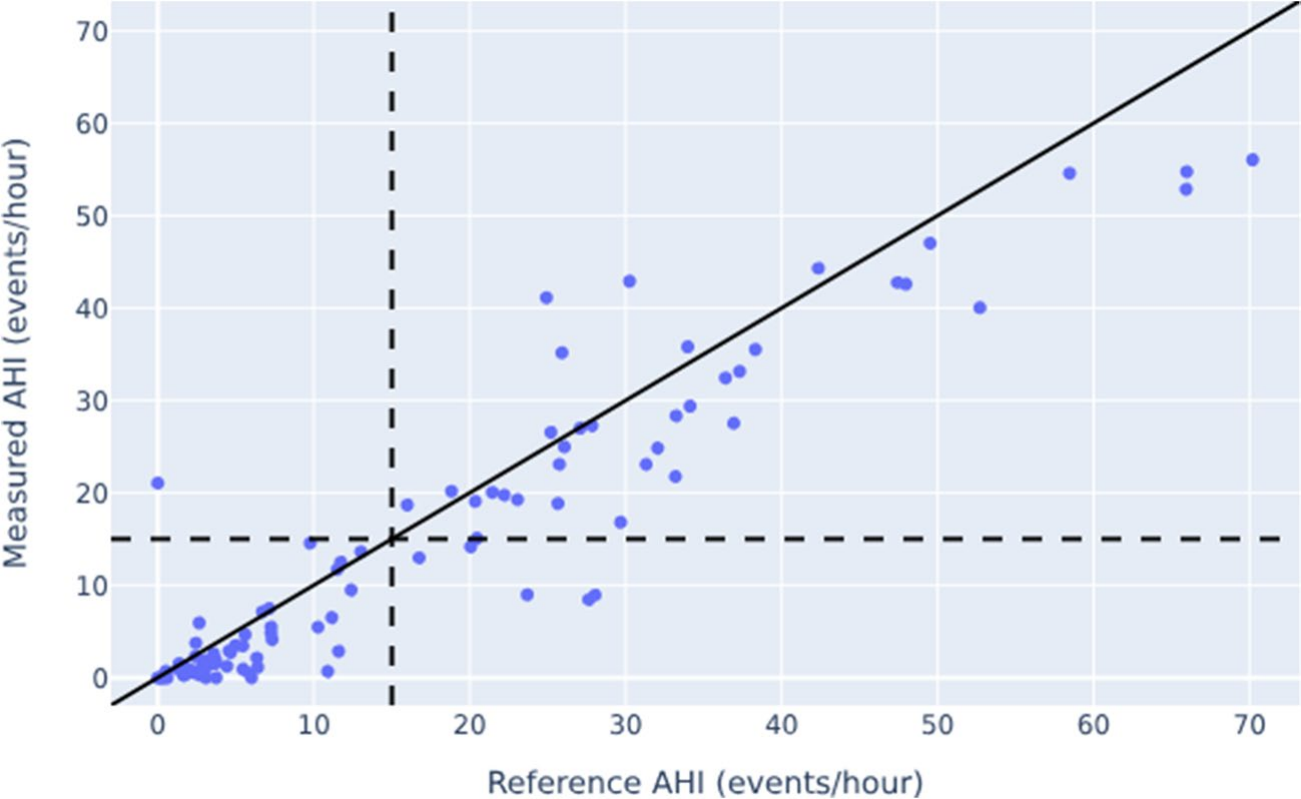
Relationship between the AHI measured by means of the Sleepiz One + monitor including an additional SpO2 sensor, and the reference AHI obtained using PSG (Please note that the black line displays the line of perfect agreement). The Pearson Correlation Coefficient between these two approaches was r_p_ = 0.94 (*p* < 0.0001). AHI: Apnea Hypopnea Index; PSG: Polysomnography; SpO2: Peripheral oxygen saturation

Correlation between the automatically determined AHI of the Sleepiz One + device and the manual AHI scoring derived from PSG was r_p_ = 0.87 (*p* < 0.0001; Fig. 2), indicating a strong linear association between both measuring systems. By adding a pulse oximeter, the correlation coefficient increased to r_p_ = 0.94 (*p* < 0.0001; Fig. 3).

**Fig. 4:**
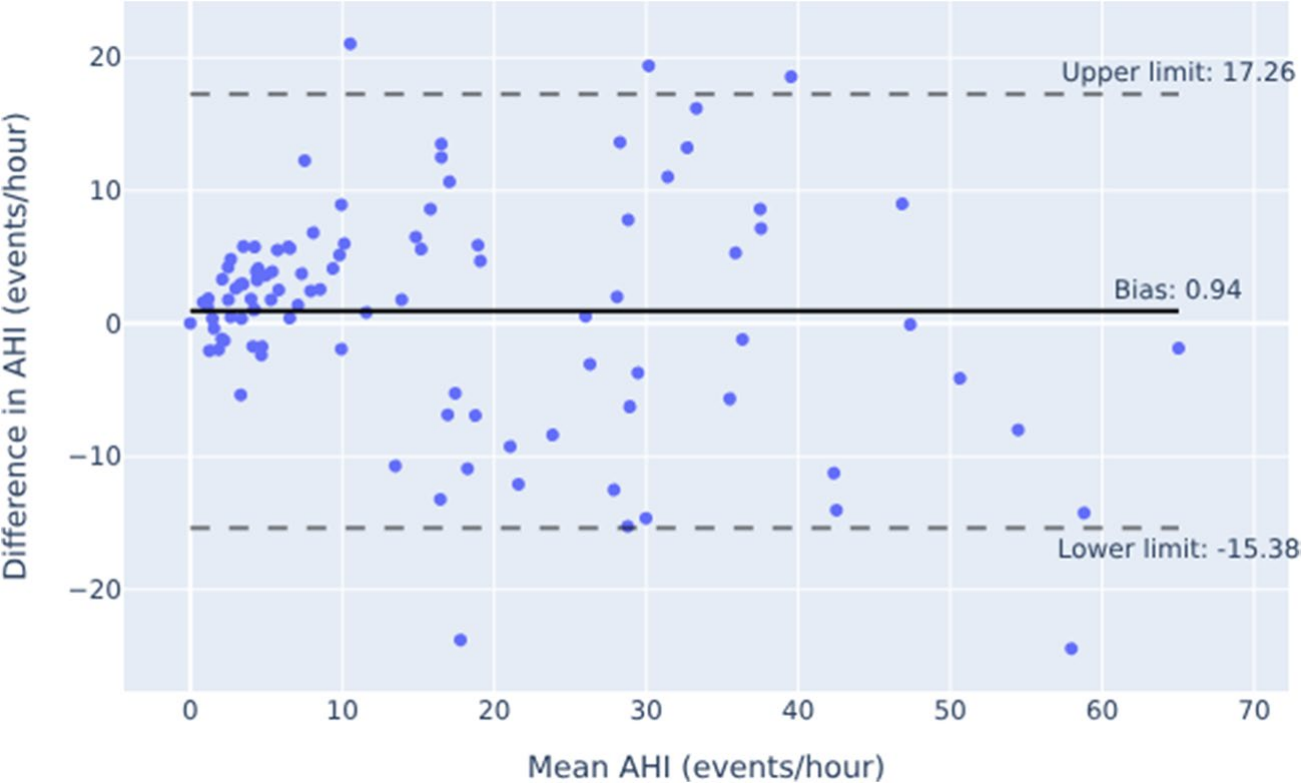
Bland–Altman Plot showing the relationship between the difference of AHI determined by Sleepiz One + and PSG (Sleepiz minus PSG), and the average of both devices. AHI: Apnea Hypopnea Index; PSG: Polysomnography

**Fig. 5:**
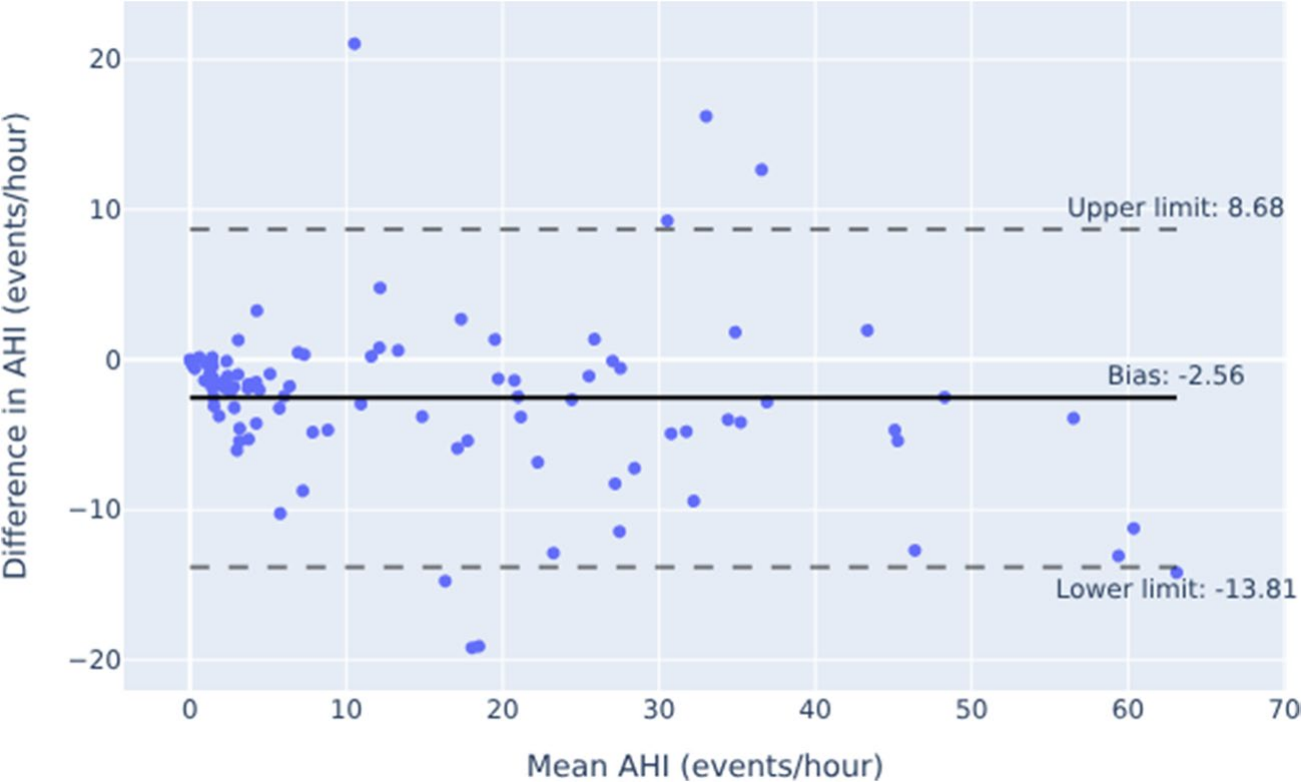
Bland–Altman Plot showing the relationship between the difference of AHI determined by Sleepiz One + with an additional SpO2 sensor and PSG (Sleepiz minus PSG), and the average of both devices. AHI: Apnea Hypopnea Index; PSG: Polysomnography; SpO2: Peripheral oxygen saturation

The Bland–Altman plots in Figs. 4 and 5 showed overall good agreement between the two measurement methods, particularly at smaller AHI values. Nevertheless, the scatter in Fig. 4 revealed some proportional disagreement, expressed by a negative correlation between the differences and the mean of the two methods (r = -0.22, *p* = 0.027). Furthermore, the widely separated limits of agreement in Fig. 4 suggested some random deviation. On the other hand, no systematic bias was present in the data as the value of 0.94 did not differ significantly from zero as demonstrated by the result of the one sample t-test (*p* = 0.262). By adding a pulse oximeter, the scattering in particular for AHI values < 10 decreased, resulting in narrower limits of agreement. Otherwise, the distributions of measurement with and without additional SpO2 show a similar pattern. Thus, the proportinal bias remained largely unchanged displaying a negative relationship between the differences and the mean of the two methods (r = -0.23, *p* = 0.0188) yet again. However, a systematic error was observed as the fixed bias of -2.56 differed from zero (one-sample t-test: *p* < 0.0001).

## Discussion and conclusions

In this study we aimed at comparing a novel contactless radar-based device for obstructive sleep apnea diagnostic against the current gold standard, the cardiorespiratory PSG. While PSG provides on the one hand reliable vital sign monitoring, it is on the other hand an inconvenient, expensive, and scarcely available resource. Treatment of OSA depends on the severity of the sleep-related breathing indicated by the AHI and the co-existence of typical symptoms such as EDS and/or medical conditions such as cardiovascular disease. According to common diagnostic criteria, OSA requires treatment if the AHI is considered at least moderate and exceeds the value of 15, regardless of any accompanying symptoms or comorbidities. In the presence of additional health conditions, therapy should be initiated for mild OSA, which is indicated by an AHI greater than 5 [17]. As the recommendation for starting treatment is more stringent in the first condition, we chose an AHI cutoff value of ≥ 15 for sensitivity and specificity analyses conducted to evaluate the usefulness of the Sleepiz One + device in clinical practice. Our results revealed that the Sleepiz One + is good at both correctly identifying people with a moderate to severe OSA (sensitivity = 85%), and correctly excluding healthy or less affected individuals (specificity = 88%). These results suggests not only a significantly better performance in diagnosing OSA compared to other diagnostic tools such as questionnaires or prediction rules, but also a similar level of accuracy compared to diverse home testing devices regularly used in sleep diagnostics [27].

A closer look at the Bland–Altman plots confirmed the overall good agreement between the two methods of AHI measurement, although it is noticeable that lower AHI values lay closer to the line of agreement than higher AHI values. While the scatter in the present data most likely reflected deviation from the true value as a result of measurement error associated with the two methods, e.g. due to a loose belt measuring respiratory effort in the PSG or movement artifacts affecting the quality of the Sleepiz One + device's respiratory signal, this bias attenuated when SpO2 was added, as shown by narrower limits of agreement, generally indicating to a benefit of including another minimal contact device for a more precise assessment. However, the similar amount of proportional disagreement between the outcome values of the two methods suggests that this improvement in performance due to SpO2 applies more to smaller AHI values, leaving the distribution of larger AHI values unaffected. In addition, the Sleepiz One + appears to slightly, but systematically underestimate the AHI when SpO2 is added. With regard to our predefined cut-off of ≤ 15, some of the false categorizations might be due to this inaccuracy. Nevertheless, it can be concluded that the Sleepiz One + device represents a promising screening alternative in OSA diagnostics.

**Fig. 6:**
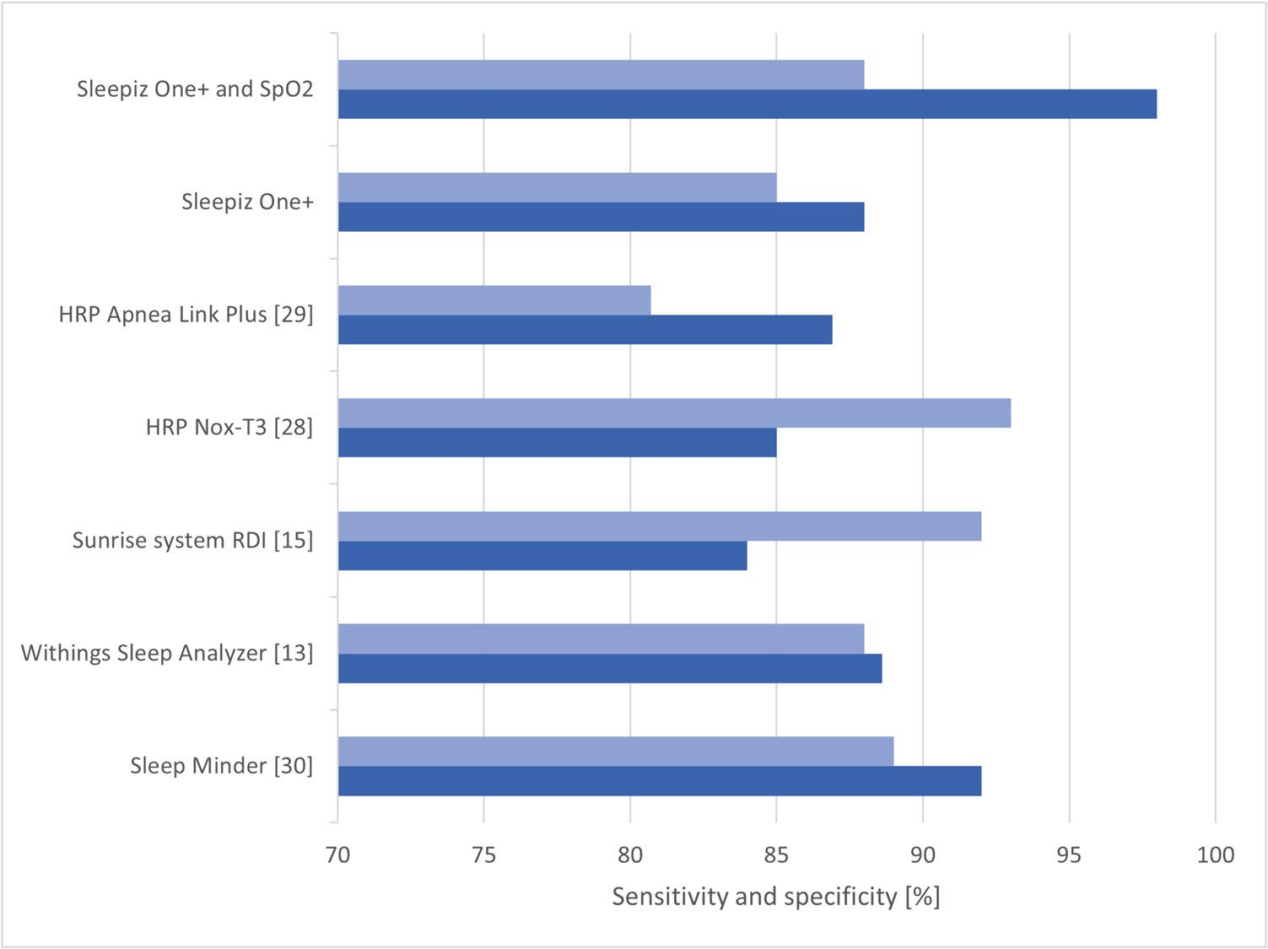
Sensitivity (upper horizontal bar) and specificity (lower horizontal bar) percentages of the Sleepiz One + monitor (with and without additional SpO2) for an AHI cutoff value of ≥ 15 in comparison to other portable OSA diagnostic devices (Sleep Minder, Withings Sleep Analyzer, Sunrise system RDI, HRP Nox-T3, HRP Apnea Link Plus with AASM scoring system). HRP: Home Respiratory Polygraph; AASM: American Academy of Sleep Medicine; AHI: Apnea Hypopnea Index; OSA: Obstructive sleep apnea; SpO2: Peripheral oxygen saturation

Further devices with little or no contact already exist on the market. A selection of these devices and their performances compared to the PSG are displayed in Fig. 6. Two Home Respiratory Polygraph (HPR) systems, tested against PSG, are also added as reference (the HRP Nox-T3-Type III Home Sleep Testing diagnostic device [28] and the HRP Apnea Link Plus, which is a portable screening device, only measuring nasal flow and desaturation [29]).

All systems showed a satisfying performance, yet surprisingly each device performs better compared to the HPR system. While the Nox HRP system demonstrated better sensitivity at the expense of specificity compared to the Sleepiz One + device, the Apnea Link Plus HRP system performed slightly worse, even when compared to Sleepiz without SpO2. This was most likely due to the different cutoff threshold, which was set to AHI ≤ 5 vs. AHI > 5 in that study. The SleepMinder is a contactless device similar to the Sleepiz One + . Thus, the similar test performance was not surprising. However, in contrast to the Sleepiz One + device, the SleepMinder is not registered as a certified medical device according to MDR [30]. The Withings Sleep Analyzer and Sunrise system RDI (Sr-RDI) are certificated medical devices but require minimal contact to the patients [13, 15]. The Sunrise system is attached to the chin of the patient measuring mandibular movements [15], while the Withings sleep analyzer is an under-the-mattress device, which measures movements of the body and chest (breathing), and vibrations due to cardiac ejection (ballistocardiography) [13]. Although all presented devices displayed roughly comparable performances, none have been implemented in clinical routine so far. HRP systems, in contrast, are generally accepted in OSA diagnostics, yet do not necessarily perform better compared to contactless or low-contact approaches including the Sleepiz One. Hence, our results are in line with those of other currently tested and available devices specifically designed for detecting OSA.

An increase in specificity due to an additional SpO2 sensor was expected and is well documented in former studies. In the HRP Apnea Link Plus validation study, the automatic assessment using only the nasal airflow signal resulted in a specificity of 37.7%, but increased to 86.9% when the automatic AASM scoring, which also included information on oxygen desaturation, was applied [29]. Thus, the additional information provided by a pulse oximeter improved specificity immensely, with only one false positive subject left, but compromised the advantage of being fully contactless. Nevertheless, adding a pulse oximeter to the Sleepiz One + still seems to offer a more comfortable solution compared to standard diagnostic such as polygraphy and PSG.

In terms of practicability and operability, Sleepiz One + offers a comfortable and easily accessible solution for sleep apnea screening. In addition, the ability to monitor vital signs in a natural environment not only provides the opportunity to track a patient's health status over time, which can help healthcare professionals make decisions about when hospitalization is advisable, but it also has the potential to improve the clinical decision process. Studies with multi-night monitoring have shown that approximately 20% of people are at risk of being misclassified in single night studies [31]. More specifically, significant night‐to‐night variability of AHI has been observed in studies of consecutive night polysomnography, with 7% to 25% of patients meeting diagnostic criteria for moderate‐to‐severe OSA the second night despite a negative result the previous night [32].

In summary, the Sleepiz One + is a non-contact screening monitor that is well suited for distinguishing between moderate and severe OSA, especially when pulse oximetry is added, as indicated by the narrower limits of agreement in the Bland–Altman plot and overall better performance results in the sensitivity and specificity analyses. Even though the Sleepiz One + device, like most other devices mentioned here, is still struggling to distinguish between sleep and wakefulness, which is an essential precondition for accurate AHI estimation, it has the potential to be a promising screening alternative in OSA diagnostics. It addresses the current lack of convenient long-term screening methods and offers an opportunity to decrease morbidity and mortality associated with OSA. The device’s automatic analysis capability, combined with the ability to conduct reliable measurements at home can save limited resources in sleep medicine facilities. However, further studies are needed to investigate the effects of long-term monitoring on disease progression in chronically ill patients. Despite these obvious advantages, it is important to emphasize that the device should only complement, but never replace the professional judgment.

### Supplementary Information

Below is the link to the electronic supplementary material.

Supplementary file1 (DOCX 10938 KB)

## Data Availability

Due to confidentiality agreements, specific datasets utilized in this research are not publicly available. Interested parties may contact the corresponding author for further information and potential collaboration.

## Declarations

### Material support

The study project was supported by the Sleepiz AG by providing the patients with Sleepiz One + devices. The authors emphasize that this support had no influence on the design, realization, or interpretation of the study.
