# Supplementary material for: Validation of the Sleepiz One + as a radar-based sensor for contactless diagnosis of sleep apnea
Source: Sleep Breath. 2024 May 14;28(4):1691–9. doi: 10.1007/s11325-024-03057-6 (PMC11303430; doi:10.1007/s11325-024-03057-6)
Supplement: Supplementary file 1 — Supplementary file1 (DOCX 10938 KB) [file 11325_2024_3057_MOESM1_ESM.docx]

## Validation of the Sleepiz One+ as a Radar-Based Sensor for Contactless Diagnosis of Sleep Apnea

**Supplements**

| **Inclusion criteria** | **Exclusion criteria** |
| --- | --- |
| - - Age ≥ 18 years   - Ability and consent to undergo electrophysiological routine assessment   - Patients suspected to suffer from sleep apnea or any other sleep-related disorder (not for healthy volunteers) | - - Previous enrollment into the current study   - Enrollment of the investigator, his/her family members, employees, and other dependent persons   - Cardiac pacemaker or another implanted electrical device   - Women who are pregnant or breastfeeding   - Inability to follow the procedures of the study, e.g. due to language problems, psychological disorders, dementia, delirium etc. of the participant   - Diagnosed or suspected sleep-related, respiratory, or cardiac disorder (only for healthy volunteers) |

Table S1: Inclusion and Exclusion Criteria.


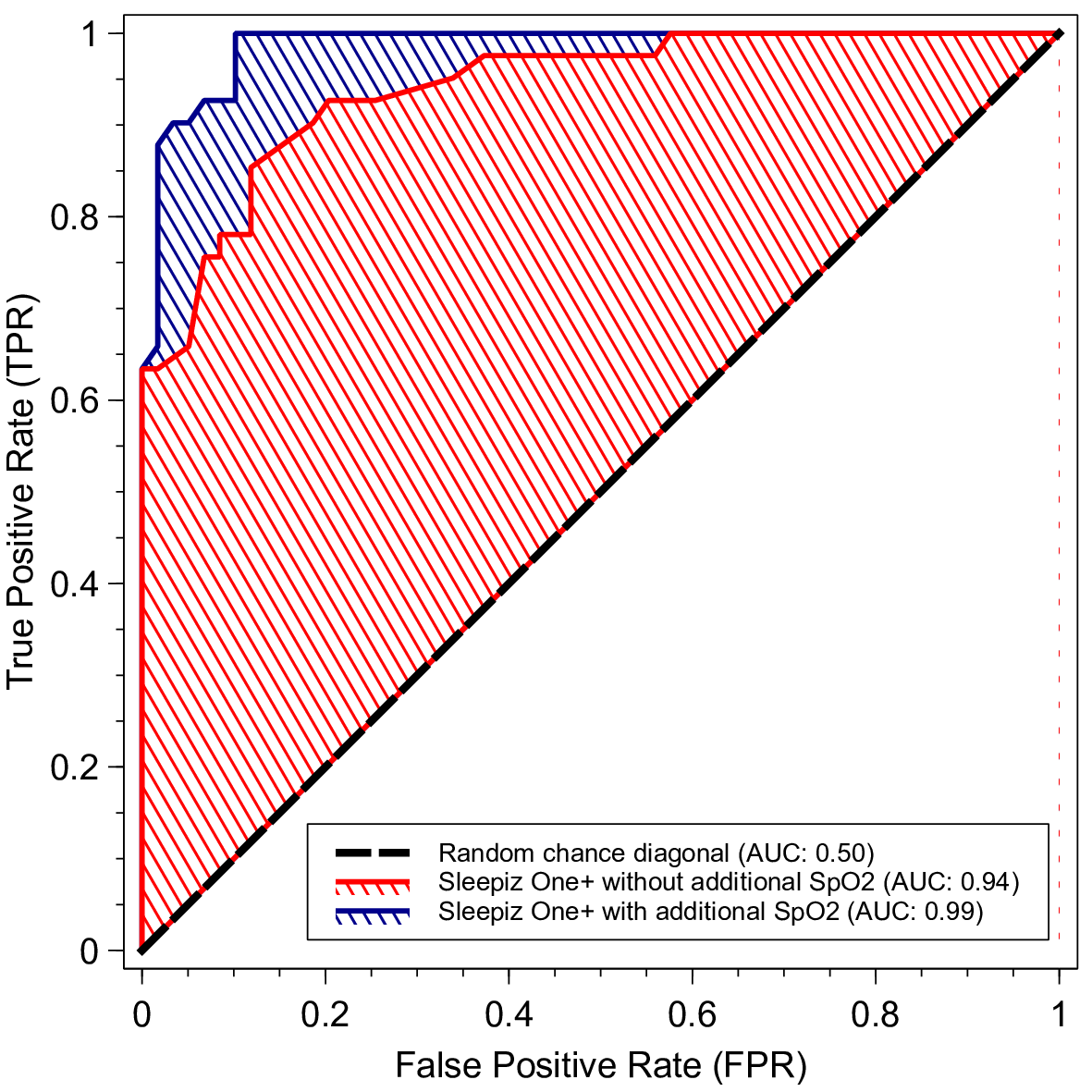


**Figure S1:** **Receiver operating characteristic curve (ROC) for the prediction of AHI based on Sleepiz+ One with (red) and without (blue) an additional pulse oximetry measurement (SpO2). The area under the curve (AUC) for measurements with SpO2 and without is 0.99 and 0.94 respectively.** SpO2: Peripheral oxygen saturation

As shown in Figure S1, the additional use of SpO2 results in a larger area under the curve (AUC) compared to the non-contact approach, indicating that the minimum contact solution has higher diagnostic accuracy.
